# Integrated arrays of air-dielectric graphene transistors as transparent active-matrix pressure sensors for wide pressure ranges

**DOI:** 10.1038/ncomms14950

**Published:** 2017-03-31

**Authors:** Sung-Ho Shin, Sangyoon Ji, Seiho Choi, Kyoung-Hee Pyo, Byeong Wan An, Jihun Park, Joohee Kim, Ju-Young Kim, Ki-Suk Lee, Soon-Yong Kwon, Jaeyeong Heo, Byong-Guk Park, Jang-Ung Park

**Affiliations:** 1 School of Materials Science and Engineering, SDC-UNIST Research Center, Ulsan National Institute of Science and Technology (UNIST), Ulsan 689-798, Republic of Korea; 2 Department of Materials Science and Engineering and Optoelectronic Convergence Research Center, Chonnam National University, Gwangju 500-757, Republic of Korea; 3 Department of Materials Science and Engineering and KI for Nanocentury, KAIST, Daejeon 34141, Republic of Korea

## Abstract

Integrated electronic circuitries with pressure sensors have been extensively researched as a key component for emerging electronics applications such as electronic skins and health-monitoring devices. Although existing pressure sensors display high sensitivities, they can only be used for specific purposes due to the narrow range of detectable pressure (under tens of kPa) and the difficulty of forming highly integrated arrays. However, it is essential to develop tactile pressure sensors with a wide pressure range in order to use them for diverse application areas including medical diagnosis, robotics or automotive electronics. Here we report an unconventional approach for fabricating fully integrated active-matrix arrays of pressure-sensitive graphene transistors with air-dielectric layers simply formed by folding two opposing panels. Furthermore, this realizes a wide tactile pressure sensing range from 250 Pa to ∼3 MPa. Additionally, fabrication of pressure sensor arrays and transparent pressure sensors are demonstrated, suggesting their substantial promise as next-generation electronics.

## Abstract (web summary)

Electronic skins and health monitoring devices rely on integrated tactile sensors, which often require tailored degrees of sensitivity in specific pressure ranges. Here, the authors fabricate a versatile matrix array of pressure-sensitive graphene transistors operating in the wide 250 Pa to 3 MPa pressure range.

Integrated electronic circuitries with pressure sensors have been extensively researched as a key component for emerging electronics applications such as electronic skins (e-skins)^1^,^2^,^3^,^4^,^5^,^6^,^7^,^8^,^9^ and health-monitoring devices^10^,^11^,^12^. Various types of pressure-sensing devices have been developed based on the sensing mechanisms of piezoresistivity^1^,^2^, capacitance^3^,^8^, piezoelectricity^10^,^12^ and field-effect transistor (FET)^4^,^5^,^11^. A piezoresistive type of device has a simple structure, high sensitivity and fast response time, but the pixel density can be low when the sensors are integrated as array forms. A capacitive type has a simple device structure and operating principles, but they are susceptible to neighbouring interference^9^. A piezoelectric type has high sensitivity and fast response time, but sensing properties can be unreliable because common piezoelectric materials also present a pyroelectric property^9^. Sensor arrays using these three types of transduction mechanism are electrically controlled by passive matrix addressing, which cannot prevent low contrast ratio and crosstalk effect. In contrast, an FET-type pressure sensor can exploit the advantages of active-matrix sensor arrays that allow high-array uniformity, high spatial contrast and facile integration with electrical circuitry. However, these FET-type pressure sensors have complicated device layouts, where additional pressure sensing components such as pressure-sensitive and conductive elastomers need to be interconnected to individual FETs and hence require relatively expensive fabrication processing costs^4^.

Here we introduce the concept of fabricating an active-matrix, pressure-sensitive graphene FET array with air-dielectric layers which are formed by folding an origami substrate, which is composed of two plastic panels and a foldable elastic joint. All electrodes of the integrated FET array can be formed together by patterning source (S)/drain (D)/interconnects on one plastic panel, and gate (G)/interconnects on the other panel simultaneously, which can simplify the overall processing steps. The elastic joint which connects these two plastic parts allows the substrate to be completely folded without any damage. The integrated arrays of top-gated transistors with local air gaps as dielectrics can be simply formed by folding this substrate to stack these two panels (one with source/drain and the other with gate). These air-dielectric graphene FETs show outstanding electrical properties and high reliability under ambient conditions, due to the clean interface between graphene channel and air. The height of air gaps is determined by the thickness of elastomeric partition spacers between the graphene and top-gate, and decreased by applying pressure with increasing capacitance of the metal-air-graphene structure. This pressure-sensitive capacitance change enables the individual FET to act as a single tactile pressure sensor solely, for a wide detection range from 250 Pa to ∼3 MPa. Therefore, the simple integration of these FETs, with no additional component or layer, forms active-matrix pressure sensor arrays directly, which can lead to low fabrication costs and densifications of these sensor arrays. Furthermore, the fabrication of a transparent pressure sensor by utilizing silver nanowire (AgNW)-graphene hybrid transparent electrodes is presented for further applications such as transparent e-skin, or an analogue touch screen panel.

## Results

### Fabrication of graphene FETs with air-dielectric layers

**Figure 1:**
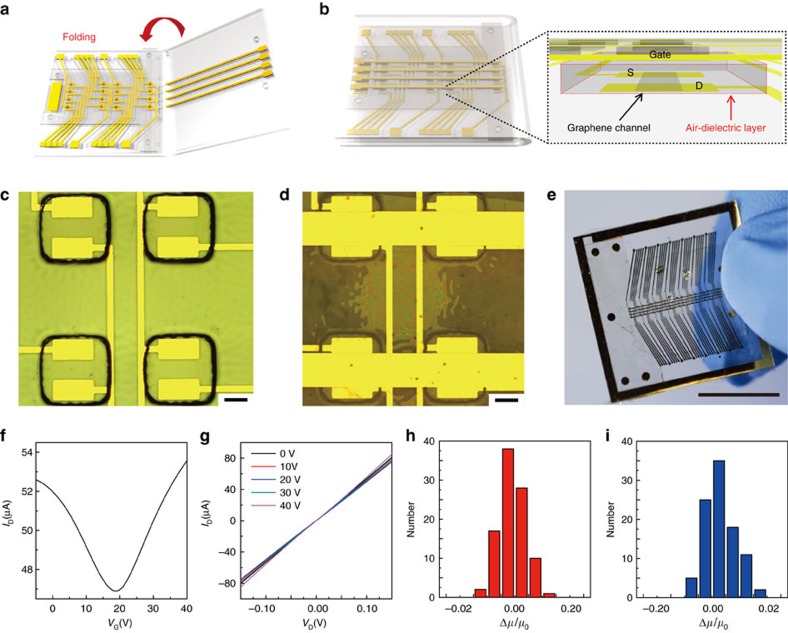
Pressure-sensitive graphene FETs with air-dielectric layers. (**a**,**b**) Schematic images of pressure-sensitive graphene FETs with air-dielectric layers before (**a**) and after (**b**) the folding, respectively. The air-dielectric layer is placed between the graphene channel and the gate electrode as illustrated in the schematic image (inset). (**c**,**d**) Optical microscopy image for graphene FETs with air-dielectric layer surrounded by p-PDMS supporting walls. Scale bars, 200 μm. (**c**) Source, drain electrodes and graphene channel are shown. Black squares are the p-PDMS supporting walls that form the air-dielectric layer. (**d**) Top gate electrodes cover the air-dielectric holes by substrate folding. (**e**) Photograph of the fabricated pressure-sensitive graphene FETs, Scale bar, 1 cm. (**f**) Transfer characteristics (*V*_D_=0.1 V). (**g**) Output characteristics (*V*_G_=0–40 V). (**h**,**i**) Statistical distribution of the relative change in the field effect mobility of the p-type (**h**) and n-type (**i**) regimes.

Figure 1a,b present the schematic illustrations of the device layout before and after folding the origami substrate, respectively. The detailed fabrication process is illustrated in Supplementary Fig. 1 and also explained in the Methods section. After forming the origami substrate composed of two plastic panels and a foldable elastic joint, graphene channels, electrodes of S/D/interconnects and elastomeric partition spacers of photo-patternable polydimethylsiloxane (p-PDMS) to define local areas of air gaps are patterned on one panel side. Also, electrodes of G/interconnects are located on the other panel. Here all electrodes of Cr (5 nm)/Au (60 nm) are simultaneously formed on both panels of the substrate using single photolithography step and metal evaporation to reduce the processing steps. The top-gate electrodes are brought into conformal contact with the p-PDMS layer and cover the top of the air-dielectric layer by folding the origami substrate in half, which completes formation of the integrated graphene FET array with local air gaps as dielectric. Patterns of positive relief with the height of ∼21.6 μm and negative reliefs with the height of ∼27.8 μm in the elastomer layer can enhance the alignment of the electrodes during this folding step, with the deviation of∼±1 μm (ref. 13). The depth of negative reliefs was made greater than the height of positive reliefs to fit with the fine aligning resolution (Supplementary Fig. 2). In addition, to highlight the novelty and merits of our device fabrication methods, we conducted the experiments for stacking integrated air-dielectric graphene FETs, which are simply formed by folding the substrate twice (Supplementary Fig. 3 and Supplementary Movie 1). Figure 1c,d show optical micrographs of the S/D, channel, and spacer in the unfolded state, and stacked top-gate FETs in the folded state, respectively. The air hole with a dimension of 600 × 600 μm, which penetrates the p-PDMS layer (thickness: 27.8 μm), is located on the graphene channel (length: 200 μm, width: 200 μm), and becomes covered by the gate electrodes in the folded state. Figure 1e shows a photograph of this integrated FET array. This array is composed of 28 FETs in total and S/D current (*I*_D_) versus top-gate bias (*V*_G_) characterization of these FETs was measured at ambient conditions. Their representative transfer and output characteristics are presented in Fig. 1f,g, respectively. The fabricated FETs show ambipolar behaviour consistent with the expected semi-metallic characteristic of graphene, with a positive charge neutrality point of ∼19 V. The statistical distribution of the relative change in the field effect mobility of p-type and n-type FETs are provided in Fig. 1h,i, respectively and these data fit Gaussian profiles. The scattering induced at oxide-graphene interfaces, such as carrier trapping, charge doping, and chemical interaction can degrade electric properties of graphene^14^,^15^,^16^,^17^,^18^, and therefore it is crucial to develop an atomically uniform gate dielectric to create a consistent electric field over the graphene in avoiding these interferences and possibly high interface defect density near Dirac point. As an example, Supplementary Fig. 4 shows transfer characteristics of SiO_2_ dielectric, representing mobility as 212 cm^2^ V^−1^ s^−1^ (p-type) and 96 cm^2^ V^−1^ s^−1^ (n-type) at *V*_D_=0.1 V. SiO_2_ can induce trapped charges at the graphene-dielectric interface or in oxide layer leading to lowering carrier mobility than that of air-dielectrics^16^,^19^. Thus, selection of the gate dielectric will be an important consideration. Although the use of suspended graphene can reduce scattering from the environments, this strategy has been limited in fabrications of only isolated, single FETs rather than integrated circuits, due to its cracking during processing steps to form the suspended structures^20^,^21^,^22^. However, utilization of folding substrates presented in this work can generate the designed arrays of local air gaps, and provide reliable integrations of graphene FET arrays with the clean interface between the air-dielectrics and graphene for high performances.

### Pressure-sensing performances of the graphene FETs

**Figure 2:**
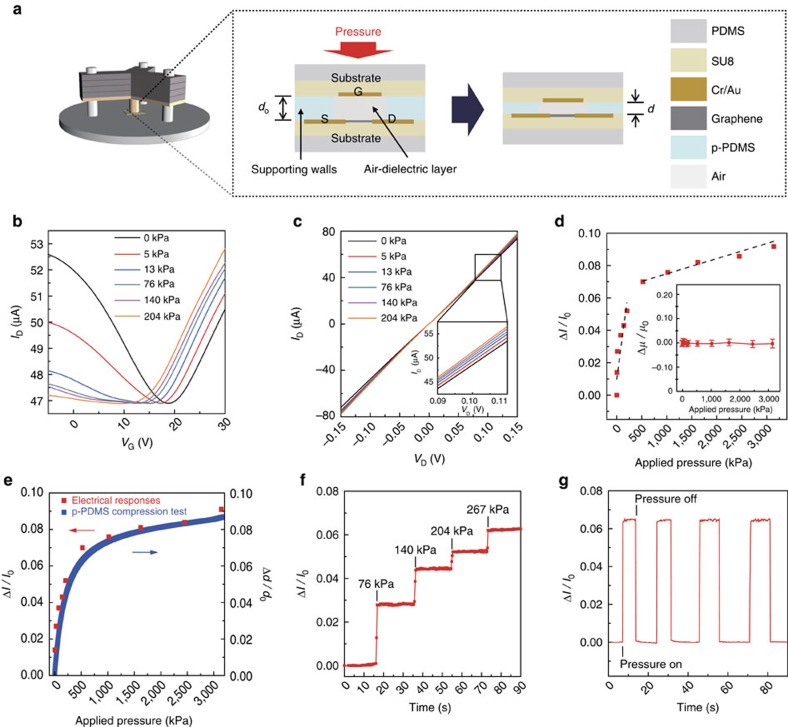
Electrical responses of graphene FETs to the applied pressures. (**a**) Schematic illustrations for the pressure sensing mechanism using an air-dielectric graphene FET. (**b**,**c**) Electrical responses of FET under different amount of applied pressures. Transfer characteristics (*V*_D_=0.1 V) (**b**). Output characteristics (*V*_G_=25 V) (**c**), respectively. (**d**) Plot of normalized drain current changes versus applied pressure. The inset illustrates relative change in the field effect mobility under applied pressure until ∼3 MPa showing almost constant values (error bars indicate the standard deviations calculated from the 1,000 times cyclic pressure loading test). (**e**) Comparison between electrical responses of the fabricated pressure sensor and true stress-strain curve from p-PDMS film compression test. (**f**,**g**) Real-time measurements of normalized drain current changes for applied pressures at *V*_D_=0.1 V and *V*_G_=25 V. The different amounts of pressure are stacked one by one sequentially representing step-like features (**f**). Pressure (267 kPa) is loaded and unloaded repeatedly to evaluate stable and reliable operation (**g**).

When the FET array is pressed by normal mechanical force, the thicknesses of the air gaps and elastomeric partition spacers (p-PDMS) decrease, with increasing the capacitance of the metal-air-graphene structure. Due to the elastic property of p-PDMS, the individual FET can act as a single pressure sensor solely with no integration of additional components or layers. Figure 2a illustrates the pressure sensing mechanism using a custom-designed tripod pressure applying machine from 5 kPa to ∼10 MPa. Transfer and output characteristics of this graphene FET, under different magnitudes of pressure, are presented in Fig. 2b,c, respectively. In both curves, *I*_D_ proportionally increases with the applied pressure at *V*_G_=25 V and *V*_D_=0.1 V. The FET shows a typical ambipolar behaviour and positive charge neutrality point of ∼19 V under no compression (black line in Fig. 2b). As the applied pressure increases; however, transconductance in p-type significantly reduces (close to zero) while the transconductance in n-type changes negligibly. This phenomenon can be attributed to the occurrence of corona discharge, as described in Supplementary Note 1. Another intriguing feature of the transfer characteristics is the left shift in the charge neutrality voltage with increasing the pressure. As the air-dielectric layer becomes thinner with resulting in a higher capacitance of the metal-air-graphene structure by increasing pressure, smaller *V*_G_ can induce the amount of charge relevant to the charge neutrality condition^23^. The plot of the normalized change in drain current (Δ*I*_D_*/I*_o_) versus applied pressure, extracted from the transfer characteristics at *V*_D_=0.1 V and *V*_G_=25 V, is shown in Fig. 2d. The detectable maximum pressure value is ∼3 MPa and Δ*I*_D_ saturates beyond this pressure range, in which the sensitivity is calculated as ∼2.05 × 10^−4^ kPa^−1^ at a lower pressure regime (below 500 kPa) and ∼9.43 × 10^−6^ kPa^−1^ at a higher pressure regime (above 500 kPa). The pressure sensor array capable of detecting the wide range of pressure has its significant importance, especially for the prospect of diverse applications. For example, the pressure sensor array in this work can be potentially used not only for the prosthetic electronic skins of robotics (from ∼10 to ∼100 kPa, which is in the range of a gentle touch to object manipulations)^10^,^24^ but also for the human weight distribution measurement during walking or clinical purposes (from 200 kPa to ∼1.3 MPa, which is in the range of non-uniform foot pressure)^25^. The sensitivity of the pressure sensor is defined as the normalized change of electrical signal per a certain amount of the pressure and it is represented in the unit of kPa^−1^ in this work. For calculation of the changes in height of air gap by the pressure, the initial thickness of the p-PDMS film was measured (Supplementary Fig. 5) and a compression test of the p-PDMS film was carried out to obtain the strain on this film for a specific pressure (Supplementary Fig. 6). True stress-strain curve was measured for this compression test, rather than that of engineering stress-strain, since the elastomer layer has a relatively high Poisson's ratio (*ν*=∼0.5) which can change the pressure-loaded area significantly when the large pressure applies. The compression test indicates p-PDMS film initially deforms in linearly elastic deformation region under low pressure below 500 kPa and presents a nonlinearly elastic deformation behaviour as the pressure increases above 500 kPa, which is the onset of nonlinearity (Supplementary Fig. 6). The thickness (*d*) of the p-PDMS film can be calculated using the equation, *d*=*d*_0_ × (1-*ɛ*), where *d*_0_ is the initial thickness of p-PDMS film and *ɛ* is the true strain of p-PDMS film for a true stress (applied pressure), which can be directly acquired from the stress-strain curve. Based on the thickness of this p-PDMS spacer, which corresponds to the thickness of the air-dielectric layer, capacitances, and mobilities of graphene FETs can be calculated (Supplementary Note 2). As presented in the inset of Fig. 2d, the graphene FETs maintain the field effect mobility with no significant change by applying pressure up to ∼3 MPa. To verify the uniform pressure distribution over the air gap even at high pressure, the air-layer thickness was measured as a function of applied pressure. Supplementary Fig. 7 shows the uniform thickness change over the whole area of air, which supports that the FET mobility can be retained even at high applied pressure. The air layer thicknesses of 112 FETs while applying the compressive pressures from 0 to 3,000 kPa was statistically investigated (Supplementary Fig. 7). This average thickness of air was compared with the air thickness obtained from the compression test of p-PDMS, and these values were very similar. Thus, it is reasonable to use the air thickness value from the compression test for calculating the device's field effect mobility using a standard metal-oxide-semiconductor FET model. To ensure the reliability and durability of the pressure sensor, a mechanical compression test of p-PDMS with a cycle of loading-unloading up to 10 MPa was carried out (Supplementary Fig. 8). Also, the repetitive compression test presents that this sensor can endure the applied compressive pressures without failures (Supplementary Fig. 9). As shown in Fig. 2e, changes in the thickness of the p-PDMS layer and air gap can modulate capacitances and therefore signals of the pressure sensor (Δ*I*_D_*/I*_o_) dominantly. As shown in Fig. 2e, changes in the thickness of the p-PDMS layer and air gap can modulate capacitances and therefore signals of the pressure sensor (Δ*I*_D_*/I*_o_) dominantly. Since this sensor operates based on the capacitance modulation proportional to the applied pressure, low-modulus dielectrics are preferred for high sensitivities. When the weights are stacked one by one, a real-time detection curve of Δ*I*_D_*/I*_o_ is shown in Fig. 2f and it represents a completely step-like features. As plotted in Fig. 2g, the recovering behaviour in pressure sensing with negligible hysteresis is exhibited during the repeated loading–unloading tests with a pressure of 267 kPa. From the four-time loading-unloading tests, the signal-to-noise ratio was calculated as ∼1,068 and the minimum pressure sensing range was estimated to be ∼250 Pa, accordingly (Supplementary Note 3). As shown in Fig. 2g, this pressure sensor operates with a response time of 30 ms and a recovery time of 52 ms (Supplementary Fig. 10). Also, the clean interface between graphene and air reduces the long-range scatterings which can lead to the negligible hysteresis behaviour of this pressure sensor.

### Active-matrix pressure-sensitive FET array

**Figure 3:**
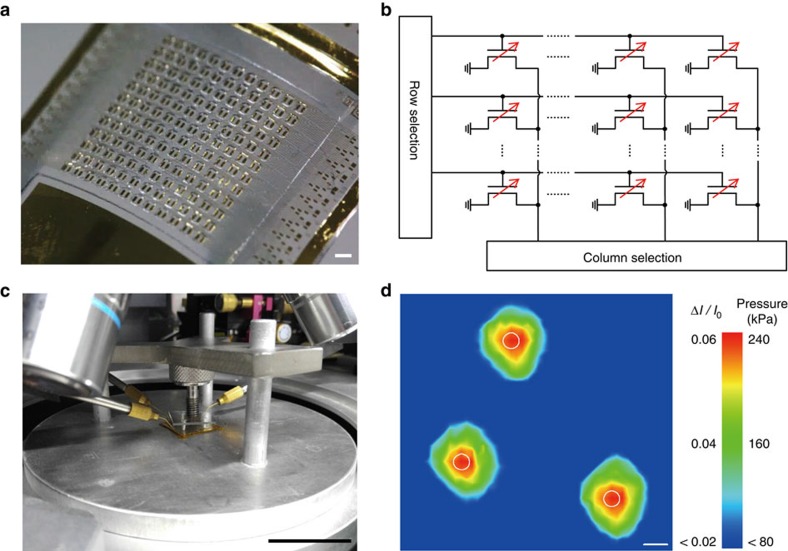
Active-matrix pressure-sensitive FET array for spatial pressure mapping. (**a**) 12 × 12 active-matrix pressure-sensitive FET array sample image before air-dielectrics are covered by top gate electrodes. The holes around source and drain electrodes which form air-dielectric layer are clearly identified. Scale bar, 1 mm. (**b**) Corresponding electrical circuitry for 12 × 12 active-matrix sensor array which allows selective readout of electrical response. (**c**) Pressure mapping set-up in probe station with the pressing machine on tripod structure which delivers the pressure to the 50 × 50 pressure sensor array. Scale bar, 5 cm. (**d**) Contour plot showing the resultant drain current change indicating how pressure was spatially distributed. White circles show the location of tripod legs and ∼240 kPa is loaded on each spot. Scale bar, 1 mm.

To exploit pressure sensors in various applications such as artificial electronic skins, touch screen panels or weight-distribution measurement devices for robotics, automotive electronics and medical diagnosis, it is necessary to fabricate integrated pressure sensor matrices with high densities of sensors. Fig. 3 demonstrates active-matrix pressure sensors using these graphene FETs with air-dielectrics. As an example, Fig. 3a shows a 12 × 12 array sample where the p-PDMS partition spacers with air holes can be clearly identified. After covering gate electrodes onto the top side of the air-dielectric layer, an integrated form of the active-matrix pressure sensor can be fabricated. In this sample, the size of individual FETs is 600 × 600 μm, providing a total distance (that is, pixel resolution) between adjacent transistors of 1 mm, which is smaller than a human's spatial resolution for of tactile sensing (1–2 mm) (ref. 26). Figure 3b illustrates the electrical circuit of this active-matrix, in which a targeted pixel can operate selectively according to the combination of row and column selection. For pressure distribution measurements, *I*_D_ was measured with biasing *V*_G_=25 V and *V*_D_=0.1 V under the pressure of approximately 240 kPa. The statistical distribution of electrical responses of 2500 FET array under applied pressure of 240 kPa is shown in Supplementary Fig. 11. In the spatial pressure mapping setup, the 50 × 50 pressure sensor array with a pixel resolution of 400 μm (Supplementary Fig. 12) was pressed down using a tripod-shaped pressing machine (column diameter: 0.8 mm), as illustrated in Fig. 3c,d presents a colour gradation contour plot of the resultant signals (Δ*I*_D_*/I*_o_), and three white circles correspond to the locations of tripod legs. Each FET of this integrated array operates as individual sensor solely with no additional component or layer, and therefore pixel resolutions of their active matrix forms can be improved further by reducing the FET size. Contrast to the previous studies using suspended gate^12^, the use of foldable substrates presented in this work can localize the array of air gaps selectively with the designed structures, and provide highly integrated active-matrix sensors with fine resolutions. The multi-touch sensing on the 20 × 20 pressure sensor array is shown in Supplementary Movie 2, and this setup is illustrated in the ‘Methods' section. Furthermore, the high pixel resolution implies the potential of integrating these pressure-sensitive FETs with other electronic components such as displaying panels or energy-storage devices for next-generation electronics.

### Fabrication of transparent pressure sensor

**Figure 4:**
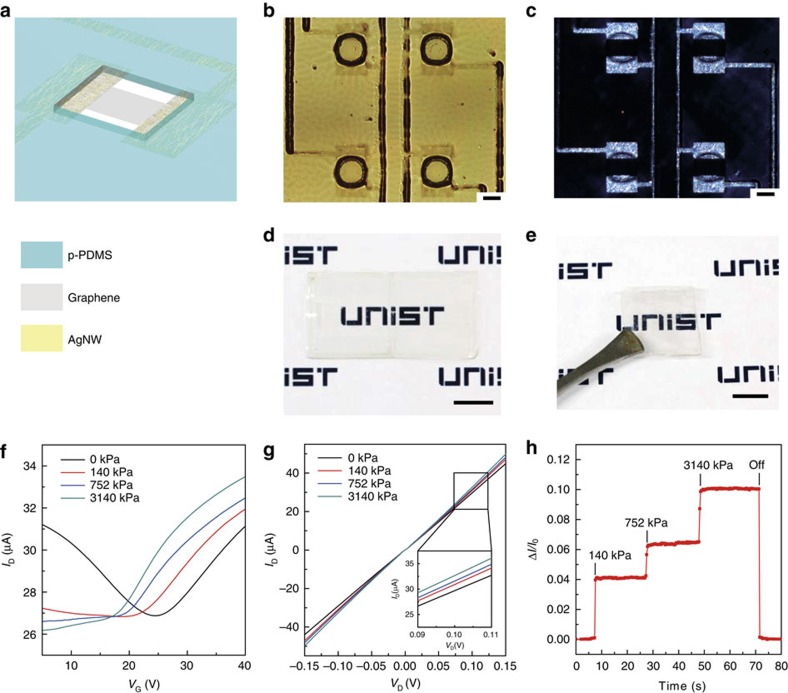
Fabrication and characterization of transparent pressure sensor. (**a**) Schematic illustration for the structure of transparent pressure sensor. (**b**,**c**) Optical microscopy images for FETs using AgNW-graphene hybrid electrodes with bright field and dark field, respectively. The holes forming air-dielectric layer can be identified in both images. Scale bars, 200 μm. (**d**,**e**) Transparent pressure sensor sample images before and after folding the substrate, respectively. The entire device including the substrate has the transmittance of ∼82% at a wavelength of 550 nm. Scale bars, 1 cm. (**f**) Transfer characteristics under different amount of applied pressure. It shows similar electrical behaviour with the non-transparent pressure sensor using Cr/Au electrodes (*V*_D_=0.1 V). (**g**) Output characteristics (*V*_G_=35 V). (**h**) Real-time measurements of normalized drain current changes for applied pressures at *V*_D_=0.1 V and *V*_G_=35 V.

Transparent forms of pressure sensor arrays can be advantageous for applications in touch screens for displays, invisible e-skins, intraocular pressure sensors, or for smart living. The fabrication of transparent, active-matrix pressure sensors by utilizing transparent electrodes using AgNW-graphene hybrid structures which allow high electrical conductance with transparency and oxidation stability^27^ is demonstrated (Fig. 4). In this approach, AgNWs were spun and photolithographically patterned. Then a chemicalvapour deposition (CVD)-synthesized graphene layer was transferred and patterned to cover the entire top surfaces of the integrated FETs where graphene connects the channel and S/D area monolithically. In this device layout, therefore, graphene and the AgNW-graphene hybrid parts serve as the channel and all electrodes of S/D/G/interconnect, respectively, as shown in Fig. 4a. The morphology characteristics of AgNW-graphene hybrid surfaces was examined through the atomic force microscopy analysis (Supplementary Fig. 13). From atomic force microscopy analysis, this hybrid structure does not present a significantly rough surface (r.m.s. roughness: ∼14 nm), which enables the integration of the hybrid parts to other electronic components. After forming the p-PDMS partition layer with air holes (Fig. 4a), the substrate was folded to stack the two panels (one with channel/source/drain and the other with gate), which completes the fabrication of transparent, integrated pressure-sensor arrays. Figure 4b,c show optical micrographs of the S/D electrodes using the hybrid with local air gaps under a bright and dark field conditions, respectively. Photos of this transparent sensor sample before and after folding the substrate are displayed in Fig. 4d,e, respectively. This transparent pressure sensor has a transmittance of ∼82% at a wavelength of 550 nm including the substrate (Supplementary Fig. 14). Transfer and output characteristics of this transparent device under different pressure levels are plotted in Fig. 4f,g, respectively. This transparent FET can also detect pressure up to ∼3 MPa, which is similar to the results of Fig. 2e. Real-time measurement of Δ*I*_D_*/I*_o_ under various pressures is presented in Fig. 4h, which exhibits the response time of 31 ms and almost complete recovery behaviour in 49 ms (Supplementary Fig. 15).

## Discussion

The integrated electronic devices with tactile pressure sensors have been widely researched for the emerging technologies such as electronic skins or health-monitoring devices. Although pressure sensors based on various transducing mechanisms were researched, it was challenging to develop integrated pressure sensor arrays with wide pressure detection ranges. This work presented the formation of active-matrix pressure-sensitive graphene FET arrays with air-dielectrics for sensing wide tactile pressure ranges. Local air spaces formed by folding the substrate induced the clean interface between the graphene channel and air, which demonstrated the integrated arrays of top-gate graphene FETs with the pressure sensitivity under ambient conditions. Also, high pixel densities and relatively fast response time are other advantages. Furthermore, the wide detection range of these pressure sensors broadens their application areas such as human touch, medical diagnosis and weight measurement for robotics, or automotive electronics. In this work, however, the large off-state current of graphene FETs is a drawback of active-matrix pressure sensor arrays using graphene FETs, which can lead to high power consumptions and cross talk. Replacing this channel material with wider bandgap semiconductors (such as organic or oxide semiconductors) or placing the graphene FETs in series with FETs with lower off-state leakages can be a potential future work for lower power consumptions and smaller cross talk. Moreover, the development and characterization of photo-patternable elastomeric materials with large toughness and small modulus as the pressure-sensing components may enhance the sensitivity of the pressure sensors while maintaining the wide pressure detection ranges. The results here give insights into innovative device geometries and suggest the promising future of next-generation electronics.

## Methods

### Preparation of substrates

Firstly, as a sacrificial layer, C4 poly(methylmethacrylate) (PMMA) (MicroChem Corp.) was spun on bare Si wafer. Then PDMS (Sylgatd 184 from Dow Corning Co.), as a soft interconnecting layer, was spun on the sacrificial layer. After the surface of PDMS was treated with reactive ion etching (RIE) system, SU8 3050 (MicroChem Corp.), as rigid islands, was spun and photo-lithographically patterned with the gap of 100 μm in order to make foldable origami substrates.

### CVD synthesis and bubbling transfer of graphene

A Cu foil (Alfa Aeasr) cleaned using acetone, isopropyl alcohol (IPA), and deionized (DI) water within the ultrasonicator (Branson 3510) was loaded into the CVD chamber. After pumping the chamber down to 10 mTorr, the furnace was heated up to 1,000 °C under 200 s.c.c.m. Ar and 500 s.c.c.m. H_2_. Graphene synthesis was carried out for 5 min under 12  s.c.c.m. of CH_4_ and 500  s.c.c.m. of H_2_. Then the chamber was cooled down to room temperature with Ar flowing at 500  s.c.c.m. and the sample was taken out from the chamber^28^. To bubbling transfer the synthesized graphene, C2 PMMA (MicroChem Corp.) was spun onto the graphene. A Cu foil with graphene and PMMA was used as a cathode and another Cu foil was used as an anode in 0.01 M KOH solution. When 4.0 V was applied by the source meter (Keithley 2400), H_2_ bubble occurred between graphene and the Cu foil, and graphene was separated from the Cu foil. The separated graphene was transferred onto the DI water using 500 μm-thickness poly(terephthalate) (PET) film and cleaned for 6 h, changing the water every 1 h. After the graphene sheet was transferred onto the target substrate and PMMA was removed by acetone which is followed by IPA and DI water cleaning^29^.

### Device fabrication

Onto the foldable substrate, Cr 5 nm/Au 60 nm are deposited using thermal evaporator and patterned by mask aligner in order to form source and drain electrodes on one side of SU8 substrate and gate electrodes on the other side. In the case of the transparent pressure sensor, instead of Cr/Au electrodes, AgNW (Nanopyxis Co. Ltd. with an average length of 30±7 μm and an average diameter of 20±5 nm) was spun on the substrate, followed by a photo-lithography process. Source and drain electrodes are comprised of AgNW-graphene hybrid electrodes. CVD-grown graphene sheet is bubbling transferred between source and drain electrodes. Graphene channel is isolated by photolithography and etch-back process with reactive ion etching system (RIE) into the dimension of 200 × 200 μm. Then, in order to form air-dielectric layer, the p-PDMS solution^30^, mixture of benzophenone (Aldrich), xylene (Samchun Pure Chemical Co. Ltd.), PDMS base, and curing agent with the ratio of 1:1:30:3, was spun onto the source-drain substrate, exposed to ultraviolet light by mask aligner, developed in toluene and uncured residue was rinsed with IPA. Then, the device was separated from the supporting Si wafer by dissolving C4 PMMA in acetone. The substrate was folded to cover the air-dielectric holes with gate electrodes and epoxy paste was applied to fix them.

### Device characterization

The electrical performances such as transfer and output characteristics of the fabricated device were characterized by probe station (Keithley 4200-SCS). To test the pressure sensing performances, pressure was applied *in situ* in a probe station using a custom-made pressure-applying machine composed of two builiding blocks. First one are the weights (50 g × 1 and 500 g × 4), and the applied weights can be from 50 g to 2.05 kg. The second one are the cylinder-shaped columns delivering pressures from the weights to the sample, which have seven different diameters of 11, 9.6, 8, 6.4, 4.8, 3.2 and 1.6 mm. Since the pressure is defined as the applied force per force-exerted area, we can apply the pressures from 5 to 10 MPa. The real-time pressure sensing experiment was carried out inside of the probe station by reading out the drain current while supplying drain and gate voltage. The spatial pressure distribution was plotted by calculating the normalized difference of electrical current between before and after applying the pressure. For multi-touch sensing on a 20 × 20 pressure sensor array, two sourcemeters (Keithley 2400), system switch (Keithley 3706), relay card (Keithley 3723), and peripheral devices were used to interconnect the pressure sensor array with processing modules. The output signal was exhibited using the Labview-based programmed software. The optical transmittance of transparent pressure sensor was measured using ultraviolet–vis spectroscopy (Agilent Cary 5000).

### Data availability

The data that support the findings of this study are available from the corresponding author upon request.

## Additional information

**How to cite this article:** Shin, S.-H. *et al*. Integrated arrays of air-dielectric graphene transistors as transparent active-matrix pressure sensors for wide pressure ranges. *Nat. Commun.* **8**, 14950 doi: 10.1038/ncomms14950 (2017).

**Publisher's note:** Springer Nature remains neutral with regard to jurisdictional claims in published maps and institutional affiliations.

## Supplementary Material

Supplementary Information Supplementary Figures, Supplementary Notes and Supplementary References

Supplementary Movie 1 3D stacking of integrated air-dielectric graphene FET array.

Supplementary Movie 2 Multi-touch sensing with 20 x 20 pressure sensor array.
