# Supplementary material for: Integrated arrays of air-dielectric graphene transistors as transparent active-matrix pressure sensors for wide pressure ranges
Source: Nat Commun. 2017 Mar 31;8:14950. doi: 10.1038/ncomms14950 (PMC5381006; doi:10.1038/ncomms14950)
Supplement: Supplementary Information — Supplementary Figures, Supplementary Notes and Supplementary References [file ncomms14950-s1.pdf]

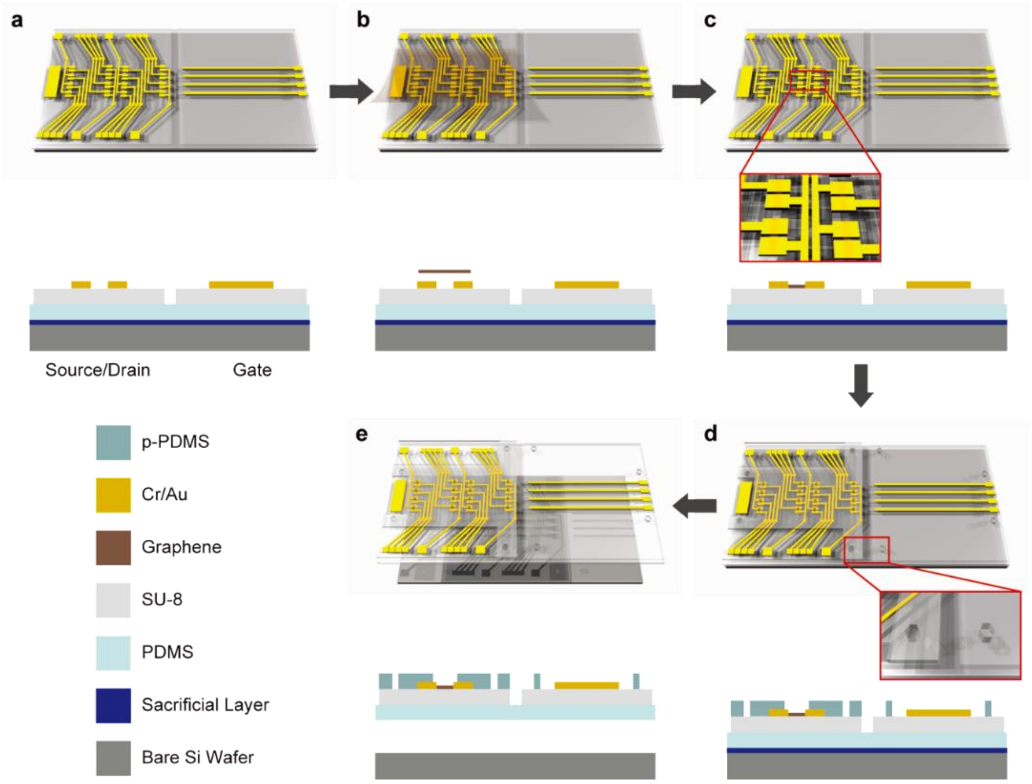

Supplementary Figure 1. Pressure sensor fabrication schematics.

Supplementary Figure 1 shows overall fabrication process and detailed illustrations are given in Methods section. (**a**) Firstly, the sacrificial layer is formed on the bare Si wafer and followed by the formation of PDMS interconnecting layer. Then the SU8 is spun and photo-lithographically patterned into the rigid panels. The Cr/Au electrodes are deposited and patterned into the source/drain on one panel and gate on the other panel. (**b**) The CVD-synthesized graphene sheet is transferred onto the panel with source/drain electrodes. (**c**) The graphene is isolated into the channels by the photolithography process. The inset shows the isolated graphene channels between the source and drain electrodes. (**d**) The photo-patternable PDMS (p-PDMS) is spun and patterned for the formation of both supporting walls for air-dielectric layers and alignment keys. The inset shows positive and negative reliefs which serve as an alignment key. (**e**) The sacrificial layer is removed and the device is detached from the handling wafer. The device is folded as shown in Figure 1a.

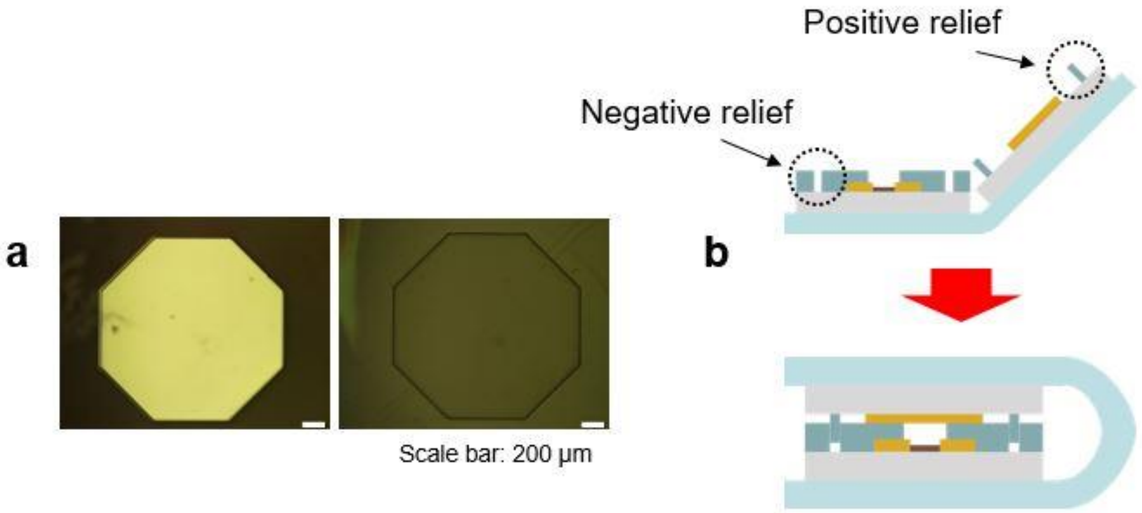

Supplementary Figure 2. Positive and negative reliefs for fine device alignment.

(**a**) The optical microscopy images of positive (left) and negative relief (right), respectively. (**b**) The schematic illustration of the self-aligning system of the foldable substrates composed of two opposing panels. Patterns of positive relief with the height of ~21.6 μm and negative reliefs with the height of ~27.8 μm in the elastomer layer can enhance the alignment of the electrodes during this folding step, with the deviation of ~ ±1 μm. The depth of negative reliefs was made greater than the height of positive reliefs to fit with the fine aligning resolution.

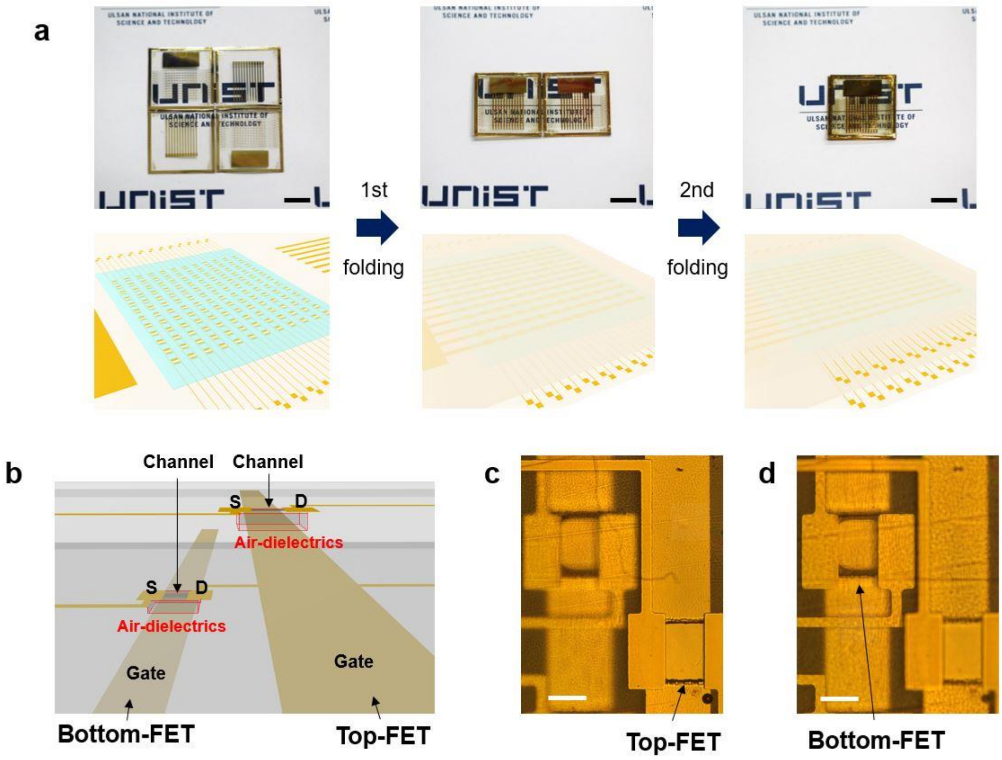

Supplementary Figure 3. 3D stacking of integrated air-dielectric graphene FET array. Double layers of the integrated air-dielectric graphene FET arrays can be three-dimensionally stacked by folding the substrate twice simply. By using alignment key comprised of positive and negative reliefs, the gate electrodes are automatically aligned. (**a**) The photographs show the sample images at unfolded state, 1st folding and 2nd folding. Scale bar, 1 cm. The schematic images illustrate the overall concept-of-proof device layout. Both top and bottom integrated FET array can be electrically biased since the tip pads are opened. (**b**) Schematics for explaining the concepts of 3D stacked top and bottom air-dielectric FET array. Source (S), drain (D), gate, channel and air-dielectrics are indicated in the schematic figure. (**c,d**) The optical microscopy images of fabricated 3D stacked integrated air-dielectric graphene FET array are presented. The devices are intentionally designed obliquely to show the 3D stacked structure. The focus is on the top-FET (**c**) and the bottom-FET (**d**), respectively. Scale bar, 200 μm.

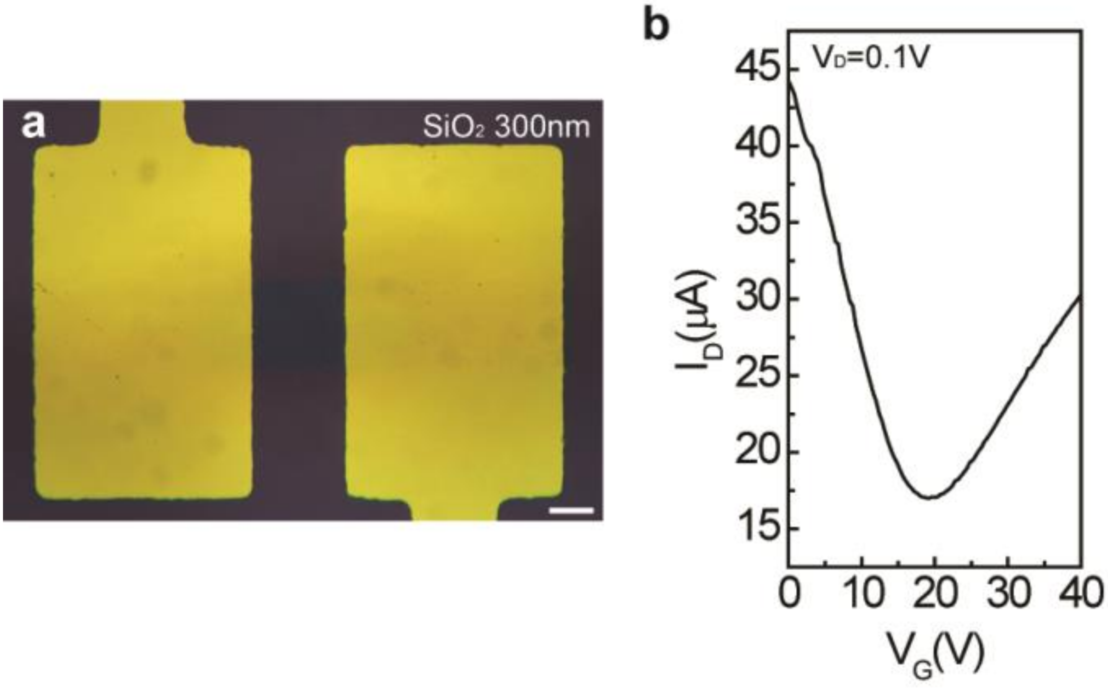

Supplementary Figure 4. The characteristics of graphene FET with SiO_2 dielectrics.

(**a**) The optical micrograph of SiO_2 dielectric (300 nm) graphene FET. Scale bar, 20 μm**.** (**b**) The transfer characteristics of the bottom-gate graphene FET. This transistor using SiO_2 dielectric has the mobility of 212 (p-type) and 96 (n-type) $cm^2\ V^{-1}\ s^{-1}$ at V_D = 0.1 V.

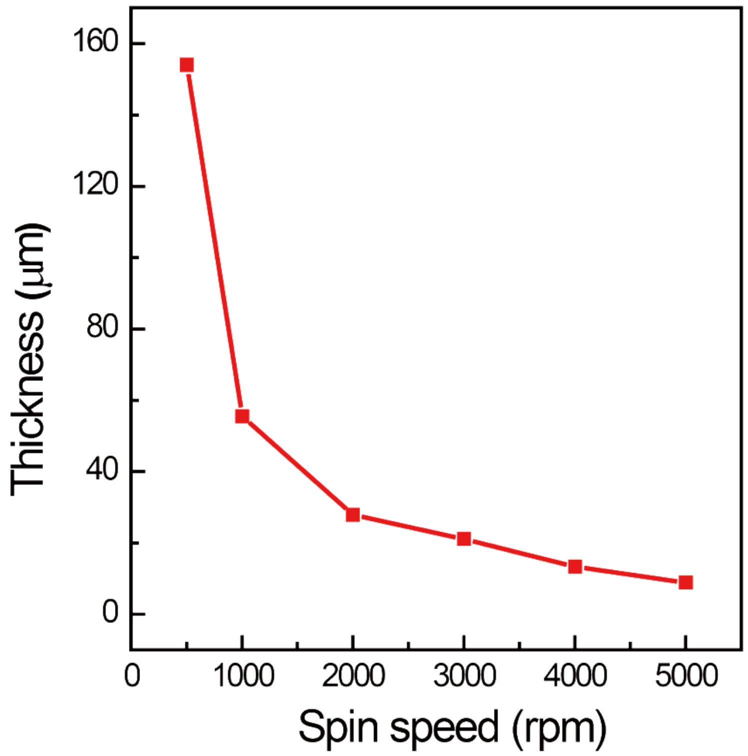

Spin speed (rpm) / thickness (μm)	500	1000	2000	3000	4000	5000
Photopatternable PDMS	154	55.4	27.8	21	13.3	8.8

Supplementary Figure 5. p-PDMS thickness measurement data for different values of spin speeds.

The thickness of p-PDMS film according to the spin speed is measured by surface profiler (KLA Tencor P6).

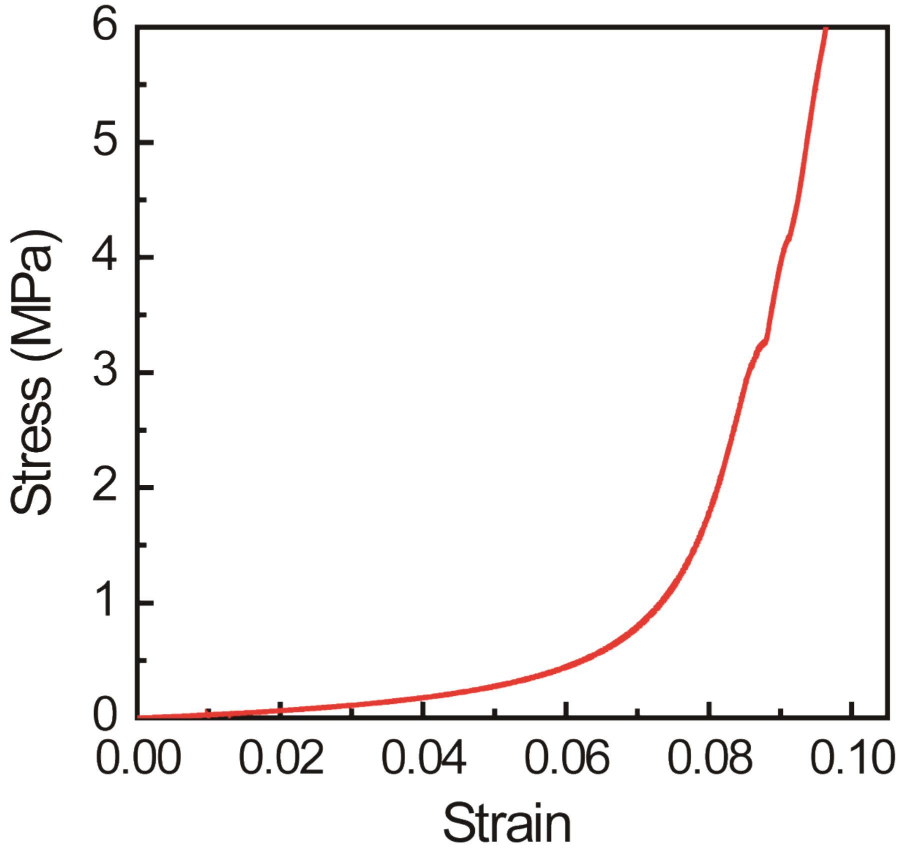

Supplementary Figure 6. True stress-strain curve of p-PDMS compression test.

The compression test of the p-PDMS film was performed in terms of true stress-strain relationship rather than that of engineering stress-strain since the elastomer has a relatively high Poisson's ratio (v = ~0.5). The compression test result indicates that the p-PDMS film initially deforms in linearly elastic deformation region and when the applied pressure reaches 500 kPa which is the onset of non-linearity, the p-PDMS film shows non-linearly elastic deformation behavior.

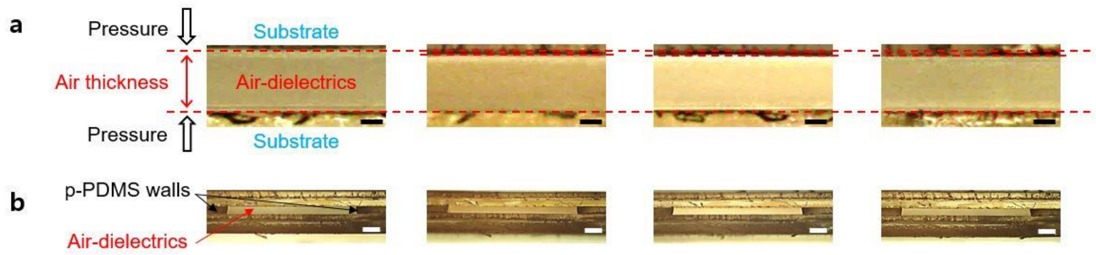

c

Applied pressure	**0 kPa**	**500 kPa**	**1500 kPa**	**3000 kPa**
(**i**) Air-dielectric thickness from compression test	27.8 μm	26.3 μm	25.8 μm	25.5 μm
(**ii**) Statistically averaged air-dielectric thickness of 112 FETs	27.78 (± 0.011) μm	26.31 (± 0.023) μm	25.80 (± 0.009) μm	25.51 (± 0.017) μm
Error rate between (**i**) and (**ii**) (|(**i**)–(**ii**)|/(**i**) × 100)	0.072 %	0.038 %	0 %	0.039 %

Supplementary Figure 7. The uniform change of air thickness under pressures.

(**a**) Optical micrographs show the air-thickness reduction with increasing the applied pressures from 0 kPa to 3000 kPa. Scale bar, 10 μm. (**b**) Optical micrographs show the uniform thickness change over the whole area of air even at a pressure up to 3000 kPa. Scale bar, 50 μm. (**c**) A comparison of the air thickness obtained from the compression test of p-PDMS with the average of air thickness measured using 112 FETs.

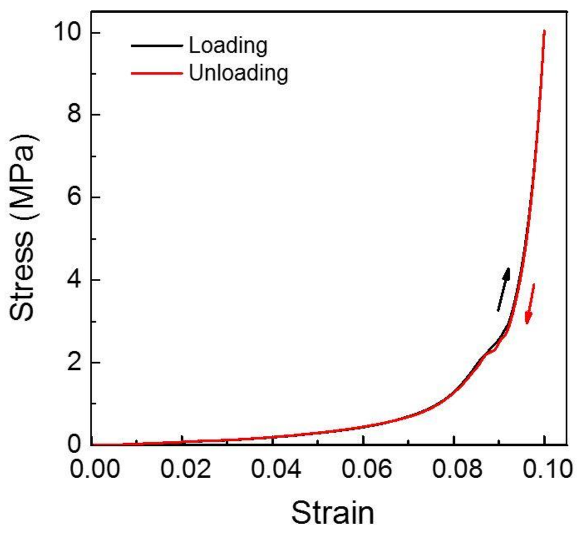

Supplementary Figure 8. Elastic property of p-PDMS demonstrated by loading-unloading compression test.

Loading-unloading compression test was implemented to prove the recovering behavior of p-PDMS (Instron Micro Tester 5948). The p-PDMS sample was compressed with true-stress up to 10 MPa and then released. The resultant data shows the elastic behaviour of p-PDMS which can endure the compressive stress up to ~10 MPa without any mechanical degradation.

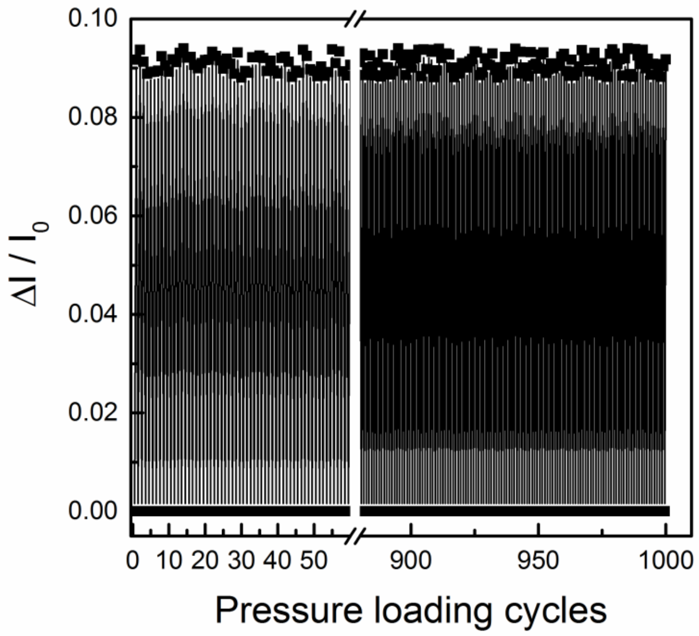

Supplementary Figure 9. Cyclic test of the pressure sensor.

For proving the reliability and durability of the pressure sensor, cyclic pressure test was conducted in which the pressure of 3.14 MPa was repeatedly loaded and unloaded for 1,000 times. During 1,000 times of cyclic test, the average value of normalized change of drain current under 3.14 MPa is calculated as 0.0910 with the standard deviation of 0.00189 at V_D = 0.1 V and V_G = 25 V. This result supports the reliability and durability of the pressure sensor even under high pressure up to ~ 3MPa.

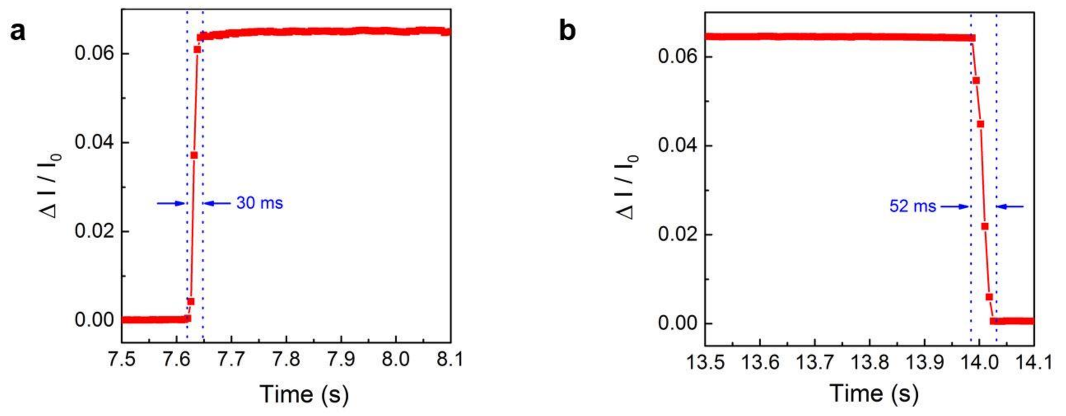

Supplementary Figure 10. The response time of the pressure sensors using Cr/Au electrodes.

Based on the real-time pressure sensing experiment given in Figure 2g, the response time and the recovery time were analyzed in detailed. The response time was measured as 30 ms (**a**) and the recovery time was measured as 52 ms (**b**).

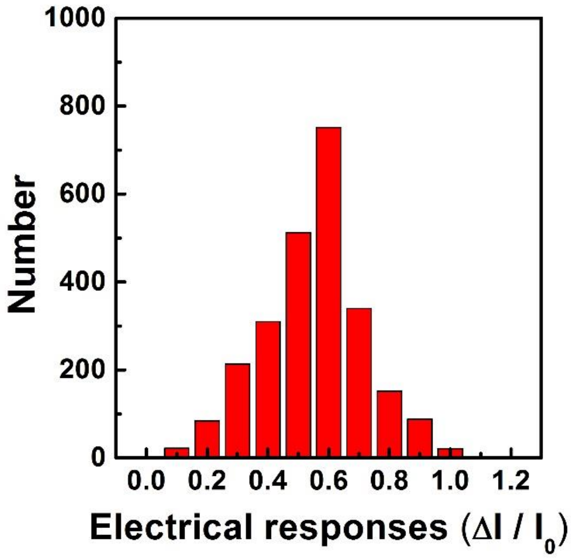

Supplementary Figure 11. Statistical distribution of electrical responses to applied pressure of 50×50 pressure sensor array.

To examine the uniformity of pressure sensor array, the statistical distribution of electrical responses of 2,500 FET array under applied pressure (240 kPa) was studied. The overall histogram data follows the Gaussian distribution with the average of 0.57 and the standard deviation of 0.23.

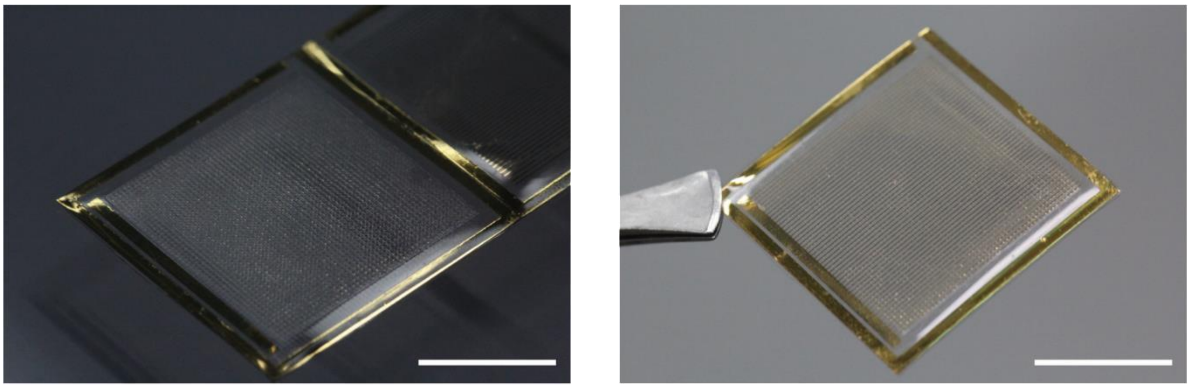

Supplementary Figure 12. 50 x 50 pressure sensitive graphene FET array.

50 x 50 pressure sensitive FET array is successfully fabricated on a couple of 2.5 ×2.5 cm^2 substrate with the resolution of 400 μm. Scale bar, 1 cm. The pixel resolution is mainly determined by the patterning feature size of p-PDMS. Holes surrounded by p-PDMS supporting walls which form air-dielectric layer are identified in the left image. The foldable substrate is completely folded and FETs are formed as top gate electrodes cover the air-dielectric layer, as shown in the right image.

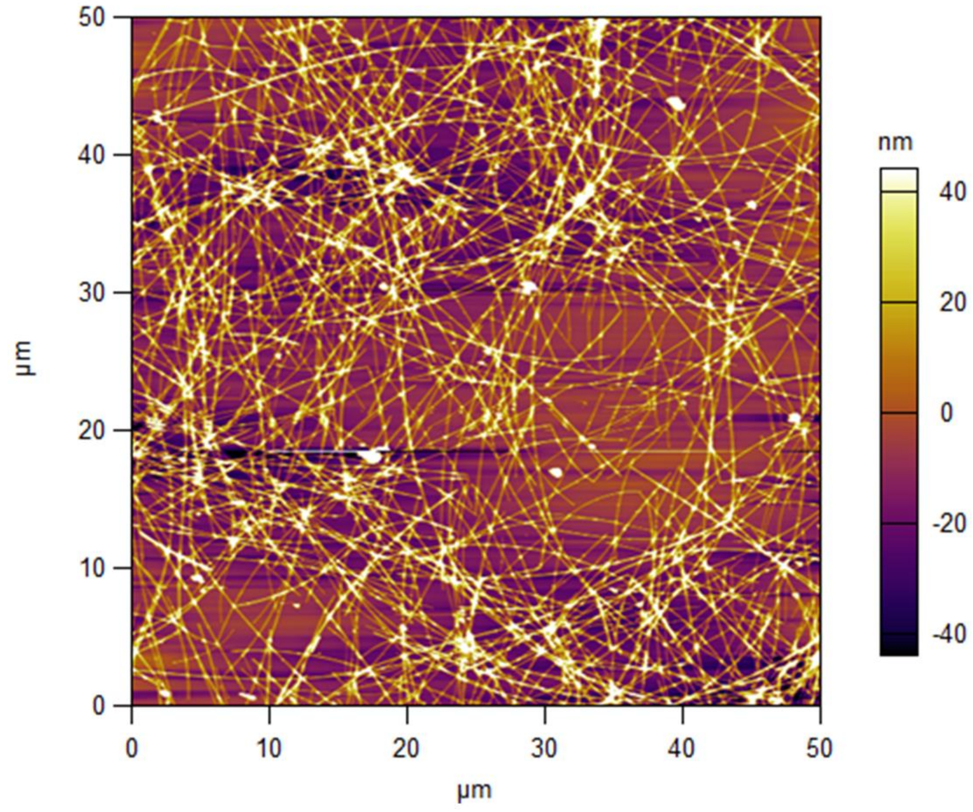

Supplementary Figure 13. Surface morphology of AgNW-graphene hybrid structure.

The morphology characteristics of AgNW-graphene hybrid structure was studied through the atomic force microscopy (AFM) analysis. In light of AFM analysis, rms roughness of the hybrid surface is ~14 nm.

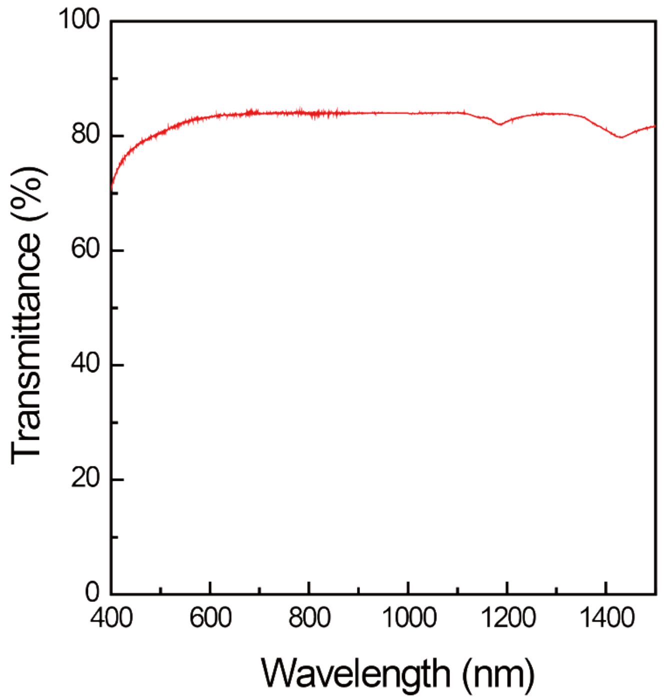

Supplementary Figure 14. Transmittance measurement of transparent pressure sensor by UV-Vis spectroscopy.

The fabricated transparent pressure sensor, including a substrate and other components as well, shows ~82% transmittance value at 550 nm.

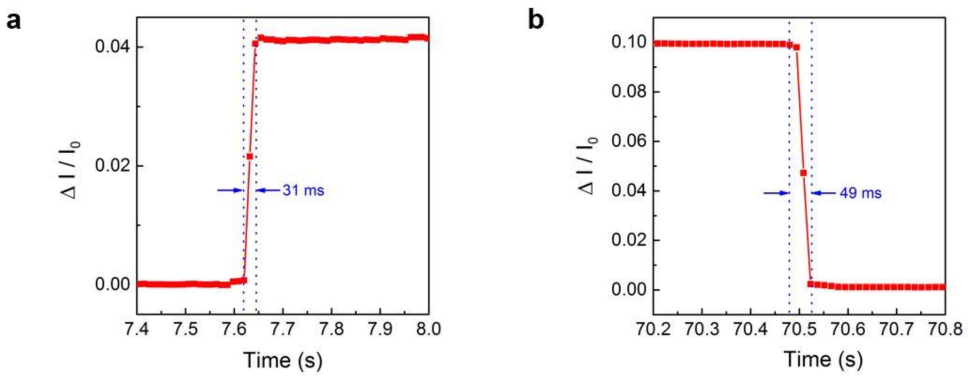

Supplementary Figure 15. The response time of the pressure sensors using AgNW–graphene hybrid structures.

Based on the real-time pressure sensing data of normalized drain current change in Figure 4h, the response time and the recovery time were measured as 31 ms (**a**) and 49 ms (**b**), respectively.

Supplementary Note 1. Occurrence of corona discharge.

Even though air is naturally an insulating layer, a corona discharge may occur when a sufficiently high electric field (> 6 V/cm)[1] is applied. When applying a negative gate bias, it has been known that the negative electric field at the air-channel interface generates negatively charged corona ions such as NO_2^- or CO_3^{2-} and these ions can trap charges in the p-type channel with degrading the channel conductance. In comparison, for the case of positive gate bias, a positive electric field at the air-channel interface forms positively charged corona ions such as $(H_2O)_n H^+$. It has been known that these ions are chemically stable relatively, and therefore their effect to the n-type channel can be less significant.

Supplementary Note 2. Mobility calculation.

Mobility of the graphene FET is calculated using below equations of standard metal-oxide-semiconductor FET model.

$$\Delta I_d = \frac{\mu \times W \times C_i \times V_D \times \Delta V_g}{L} \Rightarrow \mu = \frac{L \times {}^{\Delta I_d}/_{\Delta V_g}}{W \times C_i \times V_D} = \frac{L \times g_m}{W \times C_i \times V_D}$$

$$C_i = \frac{\varepsilon_0 \varepsilon_r}{d}$$

$$d = d_0 \times (1 - \varepsilon) = d_0 \times (1 - \frac{pressure(P)}{modulus(E)})$$

In order to derive the capacitance change, air-dielectric layer thickness is calculated based on true stress-strain curves from p-PDMS film compression test (Supplementary Figure 6).

Supplementary Note 3. Minimum pressure sensing level calculation.

As briefly illustrated in the main text, minimum pressure sensing level was calculated based on signal-to-noise ratio (SNR) of real-time measurements of normalized drain current changes (at V_D = 0.1 V and V_G = 25 V) while the pressure (267 kPa) was loaded and unloaded repeatedly for four times. SNR is a parameter for distinguishing the valid sensing results from the circumstance noise, thus the calculated SNR could be the ratio of experimentally applied pressure to the minimum pressure sensing level. SNR is defined as, SNR = P_{signal} / P_{noise} = μ/σ, where P_{signal} and P_{noise}, are average power of signal and noise, respectively, μ is the averaged value of drain currents when the pressure (267 kPa) is loaded for four times, and σ is the standard deviation of the noise levels when the pressure is unloaded for four times. From the experimental data, μ is calculated as 6.43×10^{-2} and σ is calculated as 6.02×10^{-5}, so SNR is calculated as ~1,068 (with no unit) and minimum pressure sensing level would be 267 kPa / 1068 = ~250 Pa.

Supplementary References

1. Middleton, W. M. & Valkenburg, M. E. Reference data for engineers; Radio, electronics, computer, and communications Ch. 16 (Elsevier, 2002).
